# Endovascular treatment of middle cerebral artery aneurysms: current status and future prospects

**DOI:** 10.3389/fneur.2023.1239199

**Published:** 2023-11-15

**Authors:** Zibo Zhou, Wenjing Lan, Jinlu Yu

**Affiliations:** ^1^Department of Neurosurgery, First Hospital of Jilin University, Changchun, China; ^2^Department of Radiology, First Hospital of Jilin University, Changchun, China

**Keywords:** MCA aneurysm, endovascular treatment, prognosis, complication, review

## Abstract

Middle cerebral artery (MCA) aneurysms are complex and widely distributed throughout the course of the MCA. Various types of aneurysms can occur in the MCA. Ruptured as well as unruptured MCA aneurysms may require treatment to avoid bleeding or rebleeding. Currently, clipping is regarded as the first-line choice for the treatment of MCA aneurysms. However, endovascular treatment (EVT) is emerging as an alternative treatment in selected cases. EVT techniques vary. Therefore, it is necessary to review EVT for MCA aneurysms. In this review, the following issues were discussed: MCA anatomy and anomalies, classifications of MCA aneurysms, the natural history of MCA aneurysms, EVT status and principle, deployments of traditional coiling techniques and flow diverters (FDs), and deployments and prospects of intrasaccular flow disruptors and stent-like devices. According to the review and our experience, traditional coiling EVT is still the preferred therapy for most MCA aneurysms. FD deployment can be used in selective MCA aneurysms. Parent artery occlusion (PAO) can be used to treat distal MCA aneurysms. In addition, new devices can be used to treat MCA aneurysms, such as intrasaccular flow disruptors and stent-like devices. In general, EVT is gaining popularity as an alternative treatment option; however, there is still a lack of evidence regarding EVT, and longer-term data are not currently available for most EVT devices.

## 1. Introduction

The middle cerebral artery (MCA) is the largest and most complex cerebral artery, and it is a common site for aneurysms, accounting for approximately 20% of all intracranial aneurysms (1). MCA aneurysms occur along the course of the MCA, giving them a wide distribution; in addition, various types of aneurysms can occur in the MCA. Ruptured as well as unruptured MCA aneurysms may require treatment to avoid bleeding or rebleeding. Currently, open surgery to clip MCA aneurysms is the first-line therapy. However, endovascular treatment (EVT) is becoming an attractive alternative (2–4). MCA aneurysms tend to have a wide neck and incorporate one or both of the branch vessels, which makes the EVT difficult. EVT techniques for the treatment of MCA aneurysms vary and include traditional coiling EVT, parent artery occlusion (PAO), deployment of a flow diverter (FD), and deployment of intrasaccular flow disruptors and stent-like devices (5, 6). Since the EVT technique for the treatment of MCA aneurysms is complex, a review of existing literature is necessary.

## 2. Methodology of literature collection

**Figure 1:**
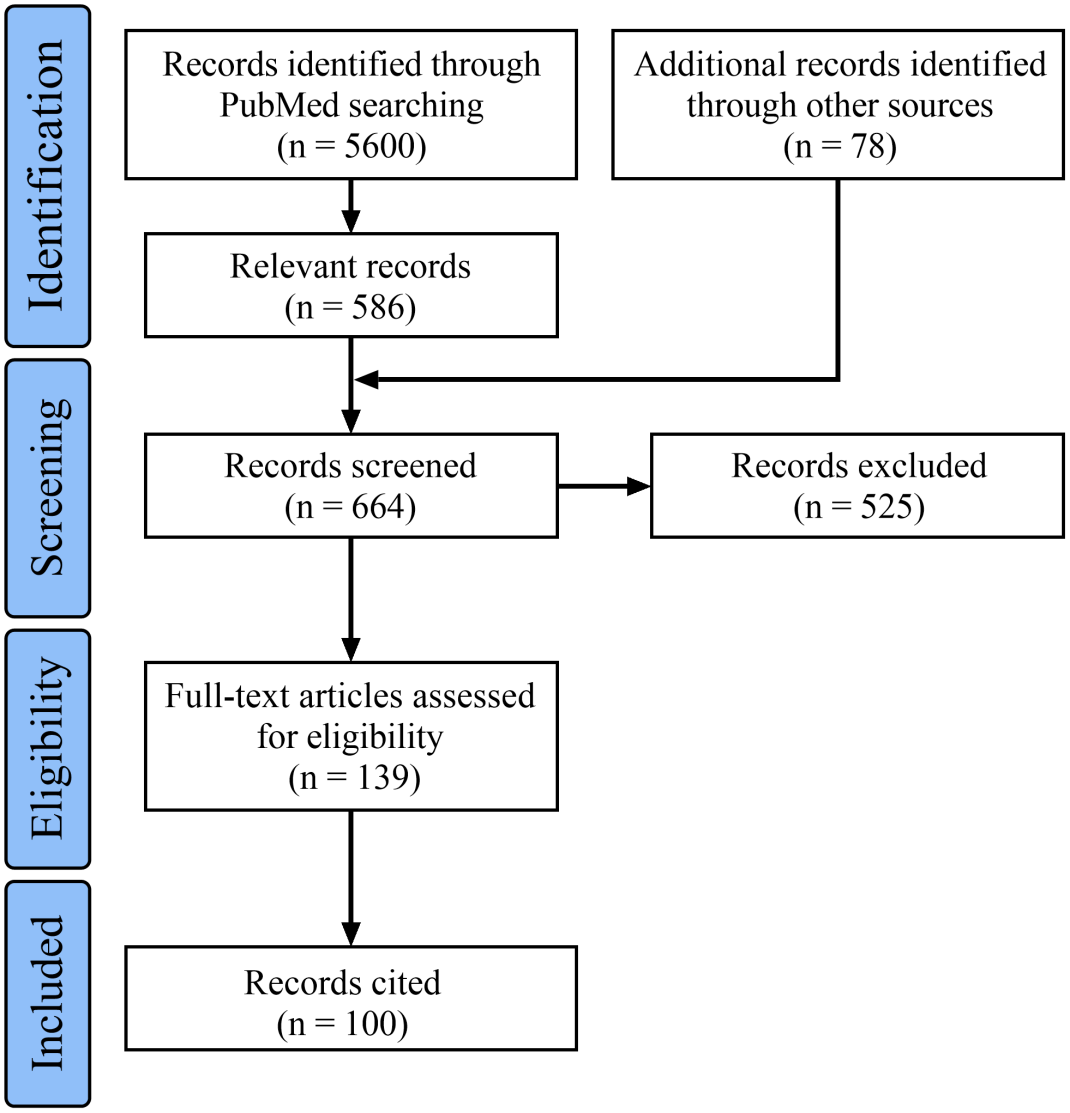
Flow chart of literature collection.

Eligible English language literature was searched in the PubMed database from 1 January 2000 to 15 August 2023. The keywords included “middle cerebral artery and anatomy, or anomaly,” “middle cerebral artery aneurysms and classification, or natural history, or endovascular treatment, or clipping, or parent artery occlusion, or flow diverter, or intrasaccular flow disruptor, or stent-like device.” A flow chart displaying the literature collection is shown in Figure 1.

## 3. Basic anatomy of the MCA

The MCA is divided into the M1 (sphenoidal), M2 (insular), M3 (opercular), and M4 (cortical) segments (7). The M1 segment belongs to the proximal part, and the M2–M4 segments belong to the distal part (8). A bifurcation-type MCA is common; single- or multiple-trunk-type MCAs are less common (7). Some cortical arteries can arise from the M1 segment, including the early temporal branch (ETB) and the early frontal branch (EFB) (9). MCA gives rise to lenticulostriate arteries (LSAs), most from the M1 segment (7, 9). MCA can have several anomalies, including fenestration, twig-like MCA, duplication, and accessory MCA (9–12).

## 4. Classifications of MCA aneurysms

### 4.1. Based on location

MCA aneurysms are commonly described in six types: (1–2) M1 bifurcation and trifurcation, (3–5) LSA, ETB, and EFB takeoff, and (6) distal MCA (13). Elsharkawy et al. simplified the classification of MCA aneurysms into proximal, bifurcation, and distal aneurysms (14). MCA bifurcation aneurysms can also include off-bifurcation aneurysms that arise within 5 mm of either side of the MCA bifurcation (15).

### 4.2. Basing morphology and pathology

MCA aneurysms can be divided into saccular and non-saccular aneurysms. Saccular aneurysms were true and tended to occur at branch takeoff from the proximal MCA and bifurcation (2). Non-saccular aneurysms were from the dissection that presented with fusiform, irregular dilation of the MCA and tended to occur at the distal MCA. MCA aneurysms may be complex, have a large or giant size, wide neck (a dome/neck ratio of <2 or a neck diameter of >4 mm), or incorporate MCA branches (16).

### 4.3. Other classifications

Based on the International Subarachnoid Aneurysm Trial (ISAT), MCA aneurysms can be divided into small (<7 mm), medium (7–12 mm), large (>12–25 mm), or giant (>25 mm) (17). There may be mirror-like MCA aneurysms (18). In addition, MCA aneurysms can be flow-related and located on feeding arteries to brain arteriovenous malformations (BAVMs) (19).

## 5. Natural history of MCA aneurysms

In Korja et al.’s study of the natural history of ruptured but untreated intracranial aneurysms, 510 patients were enrolled; 34% of patients had MCA aneurysms, and the 1-year mortality rate was 65% (20). Therefore, the risk of rebleeding in ruptured MCA aneurysms is high. The bleeding risk in unruptured MCA aneurysms increased with size (12, 21). In the unruptured cerebral aneurysm study (UCAS) in Japan, the annual rate of rupture of MCA aneurysms was 0.2% in sizes of 3–4 mm, 0.3% in sizes of 5–6 mm, 1.6% in sizes of 7–9 mm, 4.1% in sizes of 10–24 mm, and 16.9% in sizes greater than or equal to 25 mm (22). Therefore, for ruptured MCA aneurysms, and for some that are large, aggressive treatment may be necessary to avoid rebleeding and bleeding. For flow-related MCA aneurysms, the natural history is unpredictable, and due to high hemodynamic stress, they may grow (23). The size of flow-related aneurysms in supratentorial BAVMs may influence the rupture risk, especially for aneurysms with diameters ≥5 mm (24).

## 6. Endovascular treatment status and principle

### 6.1. Open surgery status

**Figure 2:**
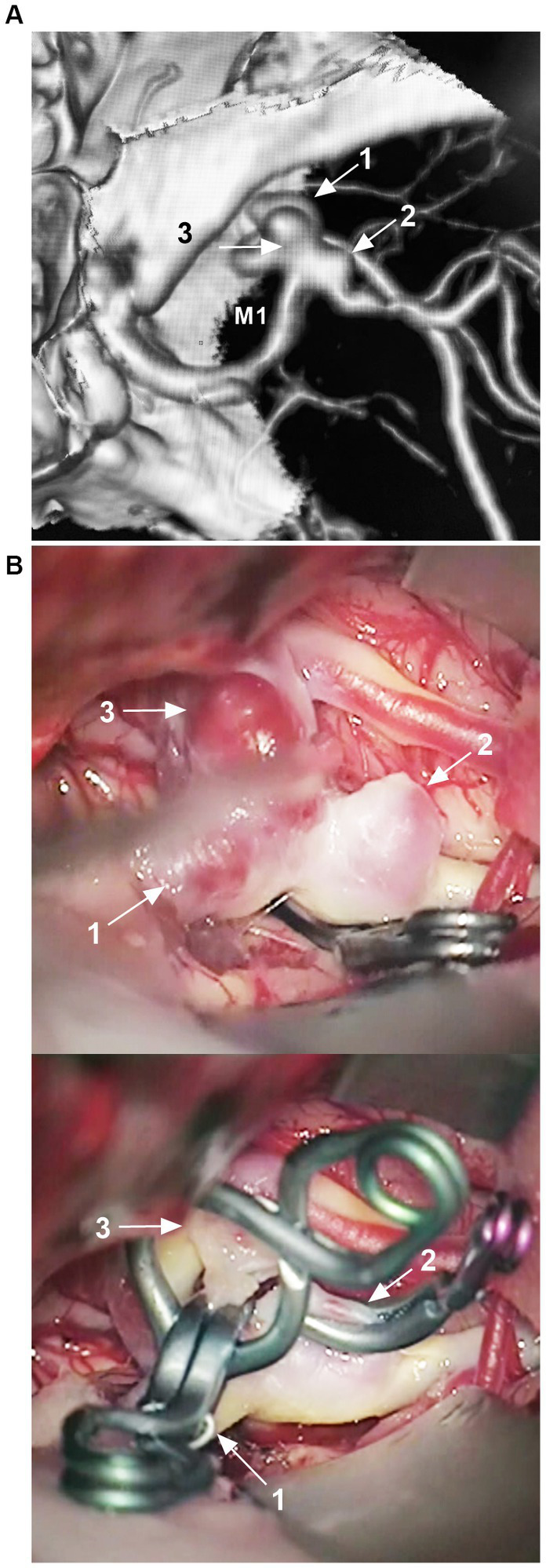
Clipping of MCA aneurysm. **(A)** CTA showing a right MCA bifurcation aneurysm, with three blebs (arrows 1, 2, and 3). **(B)** Upper panel: Intraoperative CTA image showing the aneurysm with three blebs (arrows 1, 2, and 3), MCA trunk had been clipped temporarily; Lower panel: Intraoperative image showing three blebs (arrows 1, 2, and 3) of the aneurysm that was clipped. CTA, computed tomography angiography; MCA, middle cerebellar artery; M1, first segment of the MCA.

The superficial location and features of MCA aneurysms are suitable for open surgery (Figure 2) (25). Based on a meta-analysis and clinical trials from 2015 to 2022, for MCA aneurysms, surgical clipping is still recommended first (26–29). Especially, cerebral bypass continues to be a useful tool to tackle complex MCA aneurysms (30). However, with the development of techniques and products, EVT is becoming an attractive therapy for MCA aneurysms due to its minimally invasive characteristics and safety/efficacy (31–34). In carefully selective cases, more and more MCA aneurysms can be treated by EVT (35).

### 6.2. Endovascular treatment principle

For proximal MCA aneurysms, the goal of EVT was to occlude the aneurysm and preserve the LSA, ETB, EFB, and integrated branch into the aneurysm. For distal MCA aneurysms, EVT can be performed using reconstructive or deconstructive EVT (2). For flow-related MCA aneurysms, proximal aneurysms should be treated with reconstructive EVT, and distal aneurysms can be treated with PAO (24).

## 7. Traditional EVT

### 7.1. Coiling EVT

**Figure 3:**
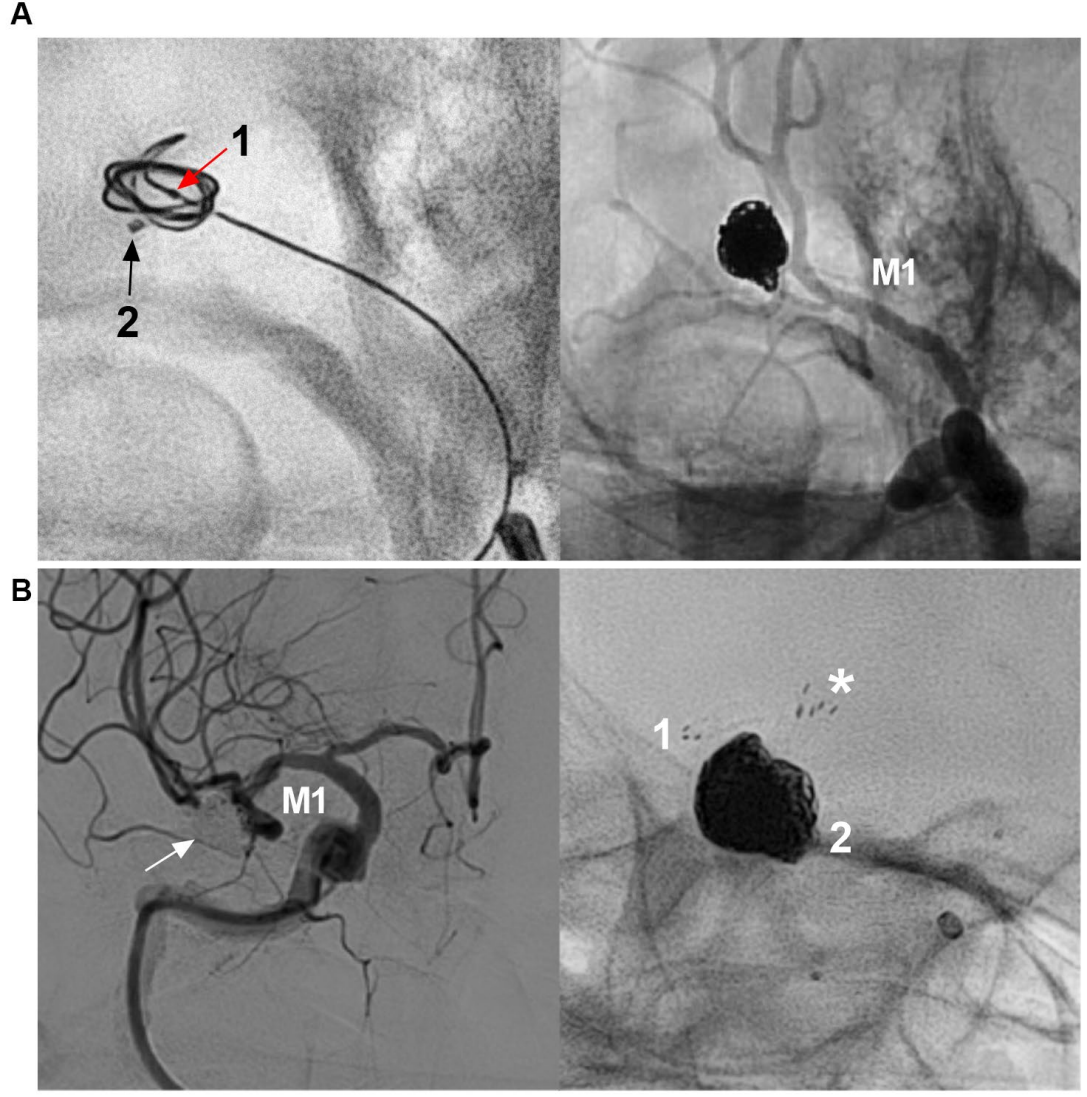
Traditional coiling EVT for MCA aneurysms. **(A)** Left panel: X-ray image showing two microcatheters into the aneurysm (arrows 1 and 2), microcatheter 1 was used for coiling first; Right panel: unsubtracted DSA showing that the aneurysm was coiled completely by the two microcraters alternately. **(B)** Left panel: DSA showing that the MCA bifurcation aneurysm (arrow) was completely coiled; Right panel: X-ray image showing that the “Y configuration” stents were used, asterisk indicated the proximal markers of the two stents. Numbers 1 and 2 indicate the distal markers of the two stents. CTA, computed tomography angiography; DSA, digital subtraction angiography; EVT, endovascular treatment, MCA, middle cerebellar artery; M1, M3, first and third segments of the MCA.

Until now, coiling EVT has remained the favorite therapy for MCA aneurysms, including dual-catheter-assisted, balloon-assisted, and stent-assisted coiling techniques (Figure 3) (32). Balloon-assisted coiling with a single microcatheter or dual microcatheter is a simple technique that offers a safe and effective solution in the management of MCA aneurysms (36, 37). Recently, the low-profile Neuroform Atlas stent (Stryker Neurovascular, Fremont, California, United States) has improved the treatment of MCA aneurysms because it allows safe catheterization of vessels as small as 1 mm (38).

**Figure 4:**
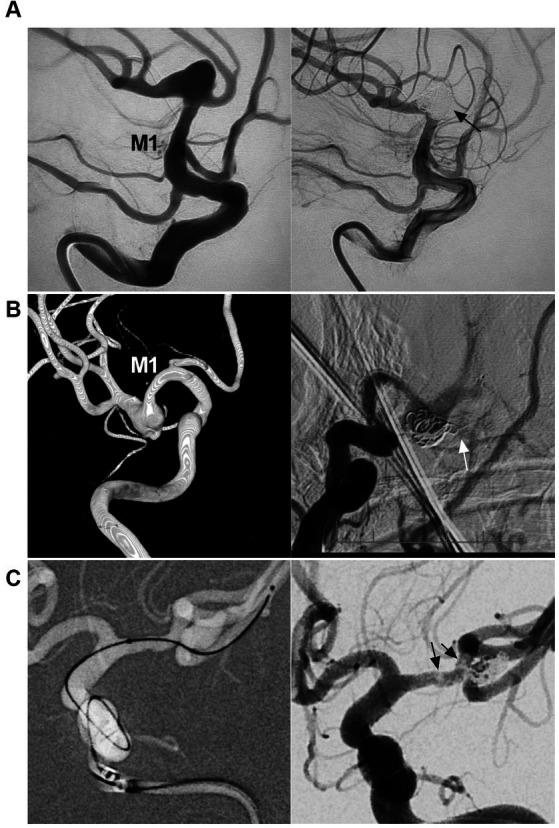
Complication of traditional EVT for MCA aneurysm. **(A)** Left panel: DSA showing a wide-necked MCA bifurcation aneurysm (arrow); right panel: DSA showing that the aneurysm was coiled with trunk occlusion (arrowhead). **(B)** Left panel: Three-dimensional DSA showing a wide-necked MCA trunk aneurysm (arrow); Right panel: DSA showing intraoperative aneurysm rupture during coiling. The arrowhead indicates active contrast extravasation. **(C)** Left panel: Roadmap image showing an MCA bifurcation aneurysm. The lower branch was catheterized to wait for stenting. Right panel: DSA showing thrombosis (arrows) in the stent during coiling of the aneurysm. DSA, digital subtraction angiography; EVT, endovascular treatment, MCA, middle cerebellar artery; M1, first segment of the MCA.

Traditional EVT for MCA aneurysms has been associated with adequate (complete occlusion and nearly complete) aneurysm occlusion and good clinical outcomes (modified Rankin Scale (mRS) score of 0–2) (27, 36). In Brinjikji et al.’s meta-analysis of 1,030 patients with 1,076 MCA aneurysms treated by coiling EVT, the morbidity and mortality rates were 5.1 and 6.0% for unruptured and ruptured aneurysms, respectively, and 82.4% of aneurysms had adequate occlusion (39). Traditional EVT for MCA aneurysms is associated with procedure-related complications (Figure 4) (2, 40, 41). EVT for ruptured MCA aneurysms had a higher rate of intraprocedural rupture. In Brinjikji et al.’s meta-analysis, the rates of intraprocedural rupture were 1.7 and 4.8% in unruptured and ruptured aneurysms, respectively (39). In Zhang et al.’s meta-analysis of 1,004 ruptured MCA aneurysms treated by coiling EVT, the overall complication rate was 22.7%, and the rates of procedure-related hemorrhagic and ischemic complications were 5 and 15.4%, respectively (42). However, most ischemic complications are asymptomatic (42). Stent-assisted coiling for EVT significantly increases the risk of procedural complications of MCA aneurysms because MCA catheterization is often difficult (12, 37).

In general, traditional EVT was appropriate for MCA bifurcation and EFB and ETB saccular aneurysms. In future, EVT can still be a useful technique for MCA aneurysms, for which the pros and cons are summarized in Table 1.

**Table 1:** Pros and cons of the main EVT techniques.

EVT type	Pro- and con
Single coiling	Single coiling EVT is the best choice for the EVT of saccular MCA aneurysm. However, even with the assistance of the dual-microcatheter technique or a balloon, the embolization may be incomplete. This technique can also be used temporarily to help the patient through the acute stage of subarachnoid hemorrhage.
Traditional stent-assisted coiling	Traditional stent-assisted coiling can improve the success rate of EVT for MCA saccular aneurysm. However, this technique increases the risk of procedure-related complications. With the development of the low-profile stent, the stent-assisted coiling technique has improved. Traditional stent-assisted coiling can be used to treat proximal MCA dissecting aneurysm but recurrence must be considered.
Parent artery occlusion	PAO in MCA aneurysm is not commonly used except for distal MCA aneurysms. PAO can result in complete occlusion of aneurysm; however, the ischemic complications must be balanced.
FD deployment	For saccular aneurysms of the LSA, ETB, and EFB takeoff, and MCA trunk dissecting aneurysm, FD deployment offers a high rate of aneurysm occlusion. However, the procedure-related ischemic complication must be considered due to the high rate of perforating arteries by the FD with a high metal coverage rate. In addition, due to no direct collateral of the MCA branch, FD deployment cannot cure all the MCA aneurysms.
Woven endobridge device	The WEB device is feasible for the treatment of MCA bifurcation aneurysms, and antiplatelet therapy is not needed. The device preserves its shape-memory property although it may not be suitable for every MCA aneurysm, and it has been used only in select cases.
pCONus device	The pCONus devices are stent-like self-expanding nitinol implants that blossom like a flower inside the aneurysm to facilitate the “waffle-cone technique.” However, there are some concerns about pCONus device deployment, especially regarding thromboembolic complications.

EFB, early frontal branch; ETB, early temporal branch; EVT, endovascular treatment; FD, flow diverter; LSA, lenticulostriate artery; MCA, middle cerebellar artery; M2, second segment of the MCA; PAO, parent artery occlusion; WEB, woven endobridge.

### 7.2. Parent artery occlusion

**Figure 5:**
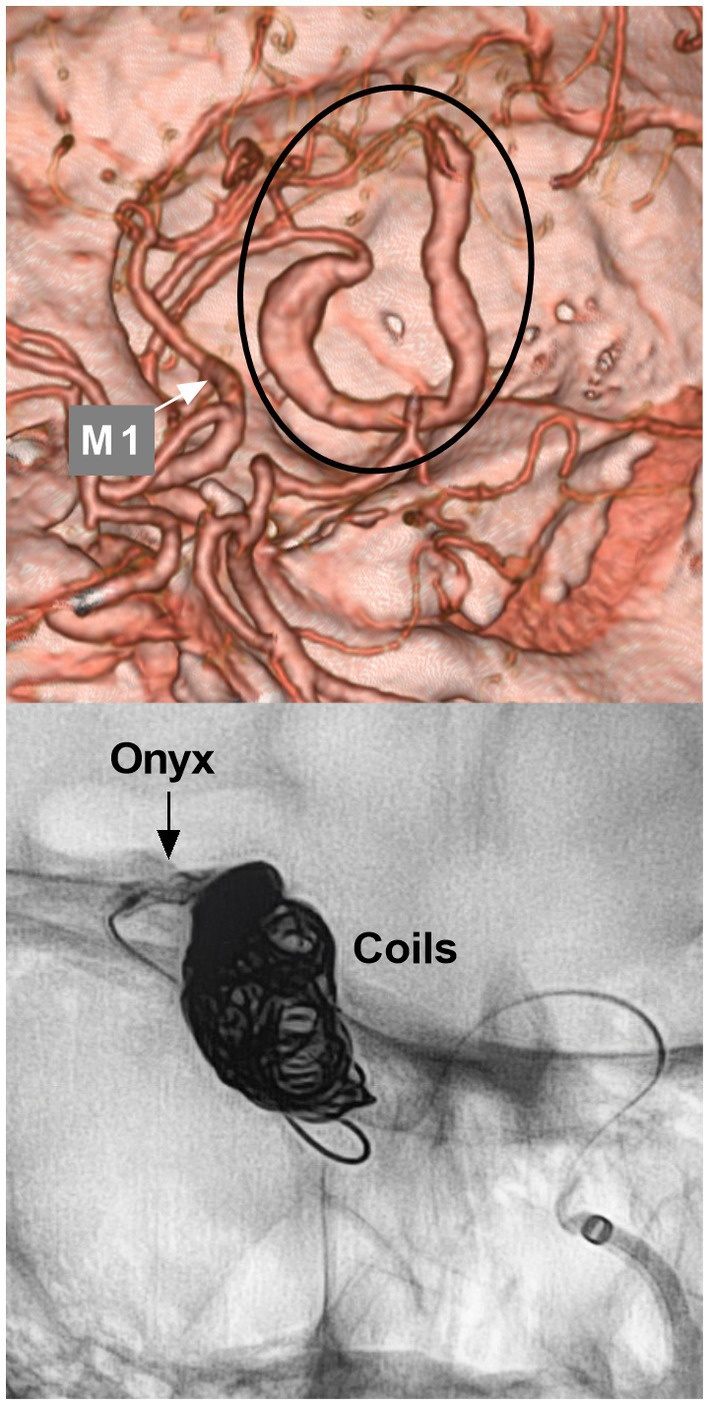
Parent artery occlusion in MCA aneurysm. Upper panel: CTA showing a serpentine aneurysm of the distal MCA; Lower panel: X-ray image showing parent artery occlusion of the aneurysm by coiling and Onyx casting. CTA, computed tomography angiography; MCA, middle cerebellar artery; M1, first segment of the MCA.

For fusiform or dissecting aneurysms of the distal MCA, PAO can still be used (43). However, PAO for MCA aneurysms should be performed cautiously (4). If the aneurysms are located on the inferior trunk of the MCA and are giant or serpentine with thrombi, the distal MCA may experience ischemic preconditioning, and branch occlusion may be safe (Figure 5) (44). PAO for aneurysms of the M3-4 segments can be performed due to adequate leptomeningeal and pial collaterals; however, this also depends on the eloquence of the affected area/branch (44). The pros and cons of PAO for distal MCA aneurysms are summarized in Table 1.

## 8. Flow diverter deployment

**Figure 6:**
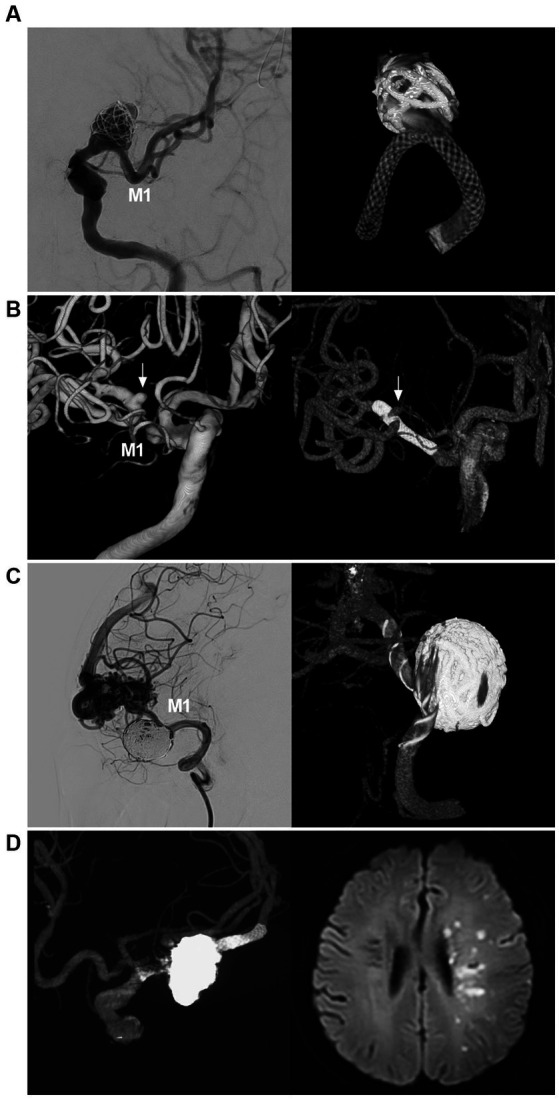
FD deployment in MCA aneurysm. **(A)** Left panel: DSA showing a wide-necked proximal MCA aneurysm (arrow); right panel: reconstructive CT showing FD deployment in the aneurysm in which the aneurysm was coiled loosely. **(B)** Left panel: Three-dimensional DSA showing an MCA bifurcation aneurysm (arrow); Right panel: Vaso-3D-DSA showing FD deployment across the bifurcation of the MCA aneurysm (arrow). **(C)** Left panel: DSA showing that a saccular flow-related aneurysm of the proximal MCA was coiled; right panel: Vaso-3D-DSA showing FD deployment in the aneurysm and that the aneurysm was coiled. **(D)** Left panel: Vaso-3D-DSA showing that an MCA bifurcation aneurysm was coiled under FD assistance; right panel: postoperative MRI showing acute ischemia of the ipsilateral hemisphere. DSA, digital subtraction angiography; FD, flow diverter, MCA, middle cerebellar artery; M1, first segment of the MCA; MRI, magnetic resonance imaging.

FD was effective for MCA aneurysms in selected cases (Figure 6) (4, 45–49). In Cagnazzo et al.’s meta-analysis (2017) of 244 MCA aneurysms that were treated by FD, 76.3% of aneurysms were located at the bifurcation or M2, aneurysms at M1-early cortical branches were 23.7%, the rate of adequate aneurysm occlusion was approximately 80%, the rupture rate per aneurysm-year was 0.4%, and the mortality rate was close to 2% (50). In Salem et al.’s multicenter cohort (2022) of 87 MCA aneurysms, good clinical outcomes were obtained in 96.8% of patients (49). The MCA aneurysm occlusion after FD deployment was progressive. Successful aneurysm healing depended on the occlusion of the branch beside the aneurysm (51).

Yavuz et al. proposed three phases of aneurysm occlusion after FD deployment, which was also appropriate for MCA aneurysms: (1) significant decrease in aneurysm filling (1–3 months), (2) infundibulum-like appearance due to the branch with a bulking origin caused by aneurysm shrinkage, the so-called “remodeled artery” (3–12 months), and (3) complete occlusion of both the covered branch and the aneurysm (6–18 months) (52). The Cekirge–Saatci grading scale can also be used to categorize MCA aneurysm occlusion: class 1: complete aneurysm occlusion, class 2: aneurysm neck filling, class 3: incomplete aneurysm filling, class 4: aneurysm filling, and class 5: stable remodeling with flow modification. When a branch comes off the aneurysm, class 1 can be subdivided: 1A as complete aneurysm occlusion with full patency of the branch, 1B as complete aneurysm occlusion with the branch reduced in caliber, and 1C as aneurysm complete occlusion with no antegrade filling of the branch (53).

Smaller bifurcating branches may exhibit less of a sump effect, remodel more rapidly, and lead to more rapid aneurysm occlusion due to low blood flow from the related branches (47, 54). Therefore, for aneurysms of the LSA, ETB, and EFB takeoff and the MCA trunk, the rate of aneurysm occlusion is high (51, 55, 56). For MCA aneurysms with a large covered bifurcating branch subjected to FD deployment, the aneurysm curative course may stop at Yavuz’s phase 1–2 or at class 3–5 of the Cekirge–Saatci grading scale (53, 57, 58). Due to the lack of direct collaterals, FD deployment does not cure all MCA aneurysms. However, reduced blood flow and aneurysm shrinkage can protect the aneurysm from rupture (52).

FD deployment in MCA aneurysms may be associated with a high complication rate (59). In Salem et al.’s multicenter cohort, the overall rates of ischemic and hemorrhagic complications were 8 and 1.1%, respectively, and symptomatic and permanent complications were encountered in 5.7 and 2.3% of patients, respectively (49). In Cagnazzo et al.’s meta-analysis, the rate of complications was 20.7%, and most were ischemic complications, often from branch occlusion and slow flow (50). To reduce or avoid ischemic complications, single FD coverage and slight oversizing were favored in consideration of decreasing mesh density to obtain slower progressive aneurysm occlusion with less risk of abrupt occlusion of the vessel coming out of the sac (53, 55). Therefore, FD may be a viable option for the EVT of MCA aneurysms. In future, with newer production developments, the safety of FD deployment in MCA aneurysms can be improved. The pros and cons of FD deployment for MCA aneurysms are summarized in Table 1.

## 9. New devices

### 9.1. Intrasaccular flow disruptors

Intrasaccular flow disruptor devices can disrupt the intra-aneurysmal flow and create thrombosis, which seems to be a promising technique for the treatment of MCA aneurysms (5, 60). Intrasaccular flow disruptor devices include the Woven Endobridge (WEB) device (Aliso Viejo, CA, United States), the Cerus Endovascular Neqstent device, Contour Neurovascular System (CNS) (Cerus Endovascular, Fremont, CA, United States), the Luna/Artisse embolization system (Medtronic, Irvine, CA, United States), and the Medina Embolic Device (MED) (Medtronic, Irvine, CA, United States) (Currently, this device is not widely available) (5, 61–63).

#### 9.1.1. Woven endobridge device

**Figure 7:**
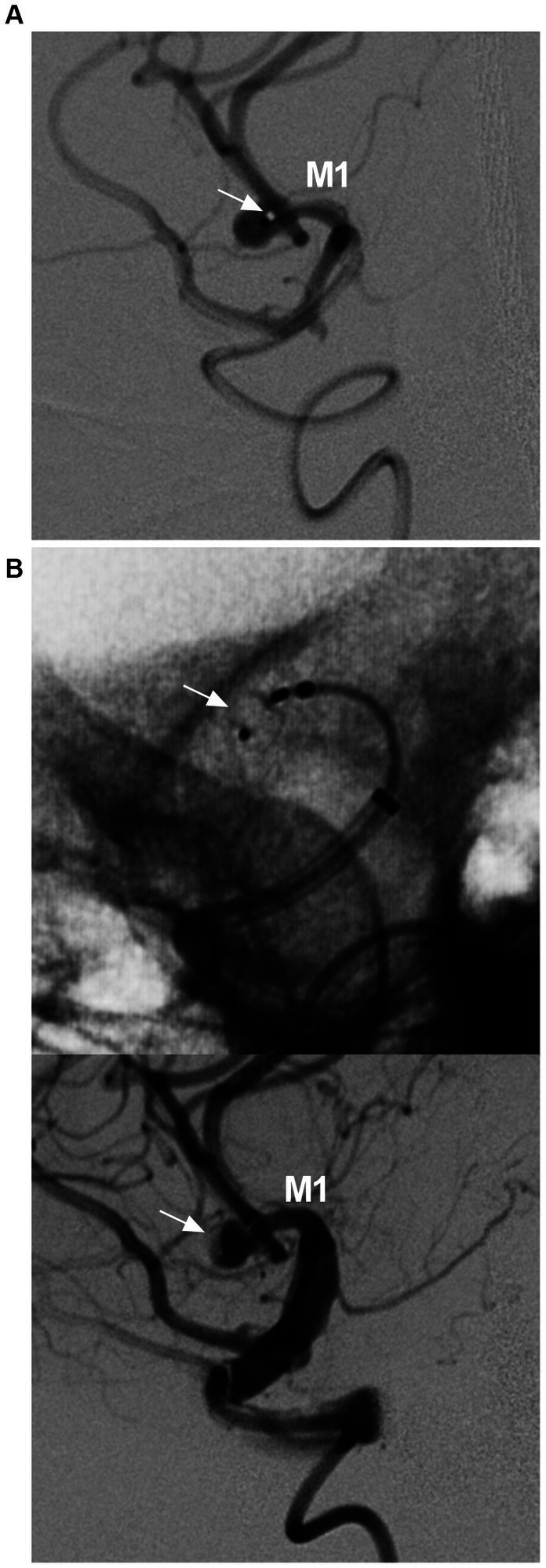
WEB device deployment in MCA aneurysm. **(A)** DSA showing the position of the VIA microcatheter (arrow) in the aneurysm. **(B)** Unsubtracted DSA (upper panel) and DSA (lower panel) showing the WEB deployed in the aneurysm (arrows). DSA, digital subtracted angiography; MCA, middle cerebellar artery; M1, first segment of the MCA; WEB, Woven endobridge.

Currently, the WEB device has evolved to a low-profile single layer with enhanced visualization (64, 65). Several landmark studies have confirmed its safety and efficacy, including WEBCAST (2016), WEBCAST-2 (2017), WEB-IT (2017), and the French Observatory (2016) (66–70). The WEB device is feasible in the treatment of MCA aneurysms (Figure 7). In particular, the current WEB-17 system is shifting its usage toward small, ruptured, and atypical aneurysms and even sidewall aneurysms (65, 71, 72).

In Adeeb et al.’s study of 206 MCA aneurysms treated by the WEB device, the adequate occlusion rate was 84.7, and 90.7% of patients had good clinical outcomes at the last follow-up (73). However, because the WEB device preserves its shape-memory property, it may not be suitable for every MCA aneurysm. Approximately 10% of WEB device-treated aneurysms may need retreatment due to compression and/or migration of the WEB device during follow-up, especially ruptured aneurysms (73–76).

The use of a WEB device with an oversized width was an independent predictor of aneurysm occlusion, and it was recommended to select a WEB device 1 mm larger than the average width and 1 mm smaller than the average height (77, 78). It is generally believed that treating the recurrence of MCA aneurysms after WEB device deployment can be more challenging. The retreatment consisted of FD deployment, stent-assisted coiling, and second WEB placement, and open surgery was the last resort (79, 80). Therefore, there is a long way to go for WEB to replace traditional EVT for MCA bifurcation aneurysms. The pros and cons of WEB device deployment for MCA aneurysms are summarized in Table 1.

#### 9.1.2. Contour neurovascular system

The CNS is a dual-layer nitinol memory mesh that provides flow diversion and flow disruption at the neck of the aneurysm. On deployment, it adapts to the lower half of the aneurysm, covering the neck (81). The CNS seems to be safe and effective in the treatment of MCA aneurysms. The success rate was >90% for the technique and > 80% for adequate aneurysm occlusion, as reported in Ghozy et al.’s meta-analysis (2022) (82), in Liebig et al.’s CERUS study (83), and in Biondi et al.’s study (84). Although good outcomes were shown, the main limitations are the small sample size and lack of long-term and randomized data.

#### 9.1.3. Neqstent device

*The* Neqstent device is a derivative of the CNS. After positioning at the neck of an aneurysm, the coiling microcatheter crosses the mesh into the aneurysm (61, 62). The Neqstent device stays within the aneurysm neck and maintains the coils within the aneurysm (85). According to the design, Neqstent can be applied to MCA aneurysms; however, reports are rare, and more experience is needed.

#### 9.1.4. Luna/Artisse embolization system

Luna named Artisse in the newest version (5, 62). Previous studies showed that the Luna/Artisse embolization system was safe and effective (86, 87). However, further study with a large sample is needed.

### 9.2. Stent-like devices

Stent-like devices include the pCONus device (Phenox GmbH, Bochum, Germany), the Barrel device (Medtronic/Covidien, Irvine, California, United States), the eCLIP (Endovascular Clip System) device (Evasc Medical Systems, Vancouver, Canada), and the PulseRider device (Cerenovus, Irvine, CA, United States) (currently, this device is not widely available) (6).

#### 9.2.1. pCONus device

Compared with other devices, pCONus devices were popularly used, including pCONus 1, pCONus 2, pCONus 2 HPC, and pCANvas devices. The pCONus devices are stent-like self-expanding nitinol implants that blossom like a flower inside the aneurysm to facilitate the “waffle-cone technique” (88–92). Some reports have shown that the use of a pCONus device to assist in coiling of MCA aneurysms successfully treats more than 95% of cases and achieves adequate aneurysm occlusion in more than 80% of cases, such as in Ulfert et al.’s report (93), in the pToWin trial (94), in Morales-Caba et al.’s report (91), and in Yeomans et al.’s report (92).

Although the above outcomes were attractive, there were some concerns about pCONus device deployment, especially regarding thromboembolic complications. In Krupa et al.’s meta-analysis of eight studies (198 patients with 200 aneurysms, including 89 MCA aneurysms), the intraprocedural complication rate was 17.3%, and the most frequent complication was thromboembolic events, occurring in 12.1% of all procedures (6). Therefore, there is a need for consensus regarding the most effective antiplatelet regimen that can be applied to reduce the incidence of thromboembolic events. In addition, more evidence is necessary to support the efficacy of the pCONus device for MCA bifurcation aneurysms.

#### 9.2.2. Barrel and eCLIP devices

The barrel device is a closed-cell microstent that can be used to reconstruct the aneurysm neck, and its key feature is a barrel section that herniates over the aneurysmal ostium (95). It can be used to assist in the coiling of MCA bifurcation aneurysms, such as in Gory et al.’s report (2018) (96) and Kabbasch et al.’s report (97). Although good outcomes were shown in the above reports, the sample size was small. A further study is needed.

The eCLIP device is a hybrid device with both neck bridging and flow-diversion properties (98). Recent studies have confirmed its effect in assisting coiling aneurysms, such as in de Vries et al.’s study (99) and in Diestro et al.’s report (100). The eCLIP device can be used for the treatment of MCA bifurcation aneurysms; however, it has been less frequently reported. More evidence is needed.

## 10. Summary

Currently, for MCA aneurysms, clipping is still regarded as the first-line therapy; EVT can be an alternative, and coiling EVT is still the preferred therapy. FD deployment can be used for the selective treatment of MCA, especially for dissecting aneurysms. PAO can be used to treat distal MCA aneurysms. In addition, some new devices can be used, such as intrasaccular flow disruptors and stent-like devices, of which the WEB device and pCONus device are advanced products. Regarding other new products for the treatment of MCA aneurysms, evidence is lacking, and longer-term data for most EVT devices are not available.

## Author contributions

JY contributed to the conception and design of the review. ZZ and WL collected data. JY and ZZ contributed to drafting the text and preparing the figures. All authors contributed to the article and approved the submitted version.

## References

[1] Inagawa T. Prevalence of cerebral aneurysms in autopsy studies: a review of the literature. Neurosurg Rev. (2022) 45:2565–82. doi: 10.1007/s10143-022-01783-7, PMID: 35460044

[2] Mortimer AM Bradley MD Mews P Molyneux AJ Renowden SA. Endovascular treatment of 300 consecutive middle cerebral artery aneurysms: clinical and radiologic outcomes. AJNR Am J Neuroradiol. (2014) 35:706–14. doi: 10.3174/ajnr.A3776, PMID: 24231847 PMC7965808

[3] Hagen F Maurer CJ Berlis A. Endovascular treatment of unruptured mca bifurcation aneurysms regardless of aneurysm morphology: short- and long-term follow-up. AJNR Am J Neuroradiol. (2019) 40:503–9. doi: 10.3174/ajnr.A5977, PMID: 30765382 PMC7028653

[4] Cimflova P Özlük E Korkmazer B Ahmadov R Akpek E Kizilkilic O. Long-term safety and efficacy of distal aneurysm treatment with flow diversion in the M2 segment of the middle cerebral artery and beyond. J Neurointerv Surg. (2021) 13:631–6. doi: 10.1136/neurintsurg-2020-016790, PMID: 33082291

[5] Heiferman DM Goyal N Inoa V Nickele CM Arthur AS. A new era in the treatment of wide necked bifurcation aneurysms: Intrasaccular flow disruption. Interv Neuroradiol. (2022):159101992210943. doi: 10.1177/15910199221094390 PMC10956467 35469513

[6] Krupa K Brzegowy P Kucybała I Łasocha B Urbanik A Popiela TJ. Endovascular embolization of wide-necked bifurcation aneurysms with the use of pCONus device: A systematic review and meta-analysis. Clin Imaging. (2021) 70:81–8. doi: 10.1016/j.clinimag.2020.10.025, PMID: 33130244

[7] Rhoton AL. The supratentorial arteries. Neurosurgery. (2002) 51:S53–S120. doi: 10.1097/00006123-200210001-00003, PMID: 12234447

[8] Rodríguez-Hernández A Zador Z Rodríguez-Mena R Lawton MT. Distal aneurysms of intracranial arteries: application of numerical nomenclature, predilection for cerebellar arteries, and results of surgical management. World Neurosurg. (2013) 80:103–12. doi: 10.1016/j.wneu.2012.09.010, PMID: 23017587

[9] Tanriover N Kawashima M Rhoton AL Jr Ulm AJ Mericle RA. Microsurgical anatomy of the early branches of the middle cerebral artery: morphometric analysis and classification with angiographic correlation. J Neurosurg. (2003) 98:1277–90. doi: 10.3171/jns.2003.98.6.1277, PMID: 12816276

[10] Hou K Xu K Liu H Li G Yu J. The Clinical Characteristics and Treatment Considerations for Intracranial Aneurysms Associated With Middle Cerebral Artery Anomalies: A Systematic Review. Front Neurol. (2020) 11:564797. doi: 10.3389/fneur.2020.564797, PMID: 33193002 PMC7654337

[11] Yu J. Current state and confusion of twig-like middle cerebral artery. Interv Neuroradiol. (2022):159101992211213. doi: 10.1177/15910199221121380 PMC11310732 35979607

[12] Zaidat OO Castonguay AC Teleb MS Asif K Gheith A Southwood C. Middle cerebral artery aneurysm endovascular and surgical therapies: comprehensive literature review and local experience. Neurosurg Clin N Am. (2014) 25:455–69. doi: 10.1016/j.nec.2014.04.005 24994084

[13] Ulm AJ Fautheree GL Tanriover N Russo A Albanese E Rhoton AL. Microsurgical and angiographic anatomy of middle cerebral artery aneurysms: prevalence and significance of early branch aneurysms. Neurosurgery. (2008) 62:ONS344. doi: 10.1227/01.neu.0000326018.22434.ed 18596514

[14] Elsharkawy A Lehečka M Niemelä M Billon-Grand R Lehto H Kivisaari R. A new, more accurate classification of middle cerebral artery aneurysms: computed tomography angiographic study of 1,009 consecutive cases with 1,309 middle cerebral artery aneurysms. Neurosurgery. (2013) 73:94–102. doi: 10.1227/01.neu.0000429842.61213.d5, PMID: 23615110

[15] Kadkhodayan Y Delgado Almandoz JE Fease JL Scholz JM Blem AM. Endovascular treatment of 346 middle cerebral artery aneurysms: results of a 16-year single-center experience. Neurosurgery. (2015) 76:54–60. doi: 10.1227/NEU.0000000000000562 25255254

[16] Zhu W Liu P Tian Y Gu Y Xu B Chen L. Complex middle cerebral artery aneurysms: a new classification based on the angioarchitecture and surgical strategies. Acta Neurochir. (2013) 155:1481–91. doi: 10.1007/s00701-013-1751-8, PMID: 23715946 PMC3718994

[17] Molyneux AJ Kerr RS Yu LM Clarke M Sneade M Yarnold JA. International subarachnoid aneurysm trial (ISAT) of neurosurgical clipping versus endovascular coiling in 2143 patients with ruptured intracranial aneurysms: a randomised comparison of effects on survival, dependency, seizures, rebleeding, subgroups, and aneurysm occlusion. Lancet. (2005) 366:809–17. doi: 10.1016/S0140-6736(05)67214-5 PMID: 16139655

[18] Zhou Z Yu J. Endovascular treatment of a supraclinoid internal carotid artery fenestration aneurysm: A case report and literature review. Heliyon. (2023) 9:e17605. doi: 10.1016/j.heliyon.2023.e17605, PMID: 37408880 PMC10318508

[19] Srinivasa R Shwetha K Pravin S Daisy S Eilene B Mathuriya SN. Microsurgical Anatomy of Middle Cerebral Artery in Northwest Indian Population: A Cadaveric Brain Dissection Study. Asian J Neurosurg. (2021) 16:785–91. doi: 10.4103/ajns.ajns_189_21, PMID: 35071078 PMC8751500

[20] Korja M Kivisaari R Rezai Jahromi B Lehto H. Natural History of Ruptured but Untreated Intracranial Aneurysms. Stroke. (2017) 48:1081–4. doi: 10.1161/STROKEAHA.116.015933, PMID: 28250196

[21] Ikawa F Morita A Tominari S Nakayama T Shiokawa Y Date I. Rupture risk of small unruptured cerebral aneurysms. J Neurosurg. (2019) 132:69–78. doi: 10.3171/2018.9.JNS181736 30684948

[22] Investigators UJ Morita A Kirino T Hashi K Aoki N Fukuhara S. The natural course of unruptured cerebral aneurysms in a Japanese cohort. N Engl J Med. (2012) 366:2474–82. doi: 10.1056/NEJMoa1113260 22738097

[23] Świątnicki W Boecher-Schwarz H Standhardt H. Growth of flow-related aneurysms following occlusion of cerebral arteriovenous malformation. J Neurol Surg A Cent Eur Neurosurg. (2023). doi: 10.1055/a-2037-6079 36808403

[24] Stein KP Wanke I Forsting M Zhu Y Moldovan AS Dammann P. Associated aneurysms in supratentorial arteriovenous malformations: impact of aneurysm size on haemorrhage. Cerebrovasc Dis. (2015) 39:122–9. doi: 10.1159/000369958, PMID: 25660640

[25] Metayer T Leclerc A Borha A Derrey S Langlois O Barbier C. Microsurgical Clipping of Middle Cerebral Artery Aneurysms: Complications and Risk Factors for Complications. World Neurosurg. (2022) 168:e87–96. doi: 10.1016/j.wneu.2022.09.044, PMID: 36115562

[26] Zijlstra IA Verbaan D Majoie CB Vandertop P van den Berg R. Coiling and clipping of middle cerebral artery aneurysms: a systematic review on clinical and imaging outcome. J Neurointerv Surg. (2016) 8:24–9. doi: 10.1136/neurintsurg-2014-011478 25431306

[27] Alreshidi M Cote DJ Dasenbrock HH Acosta M Can A Doucette J. Coiling Versus Microsurgical Clipping in the Treatment of Unruptured Middle Cerebral Artery Aneurysms: A Meta-Analysis. Neurosurgery. (2018) 83:879–89. doi: 10.1093/neuros/nyx623, PMID: 29438551

[28] Toccaceli G Diana F Cagnazzo F Cannizzaro D Lanzino G Barbagallo GMV. Microsurgical Clipping Compared with New and Most Advanced Endovascular Techniques in the Treatment of Unruptured Middle Cerebral Artery Aneurysms: A Meta-Analysis in the Modern Era. World Neurosurg. (2020) 137:451–64.e1. doi: 10.1016/j.wneu.2019.12.118, PMID: 31972346

[29] Sturiale CL Scerrati A Ricciardi L Rustemi O Auricchio AM Norri N. Clipping versus coiling for treatment of middle cerebral artery aneurysms: a retrospective Italian multicenter experience. Neurosurg Rev. (2022) 45:3179–91. doi: 10.1007/s10143-022-01822-3, PMID: 35665868 PMC9492556

[30] Shenoy VS Miller C Sen RD McAvoy M Montoure A Kim LJ. High-Flow Bypass and Clip Trapping of a Giant Fusiform Middle Cerebral Artery (M1) Aneurysm: Technical Case Instruction. Oper Neurosurg (Hagerstown). (2023) 25:e183–7. doi: 10.1227/ons.0000000000000785, PMID: 37307021

[31] Duan G Zhang Y Yin H Wu Y Zhang X Zhao R. Predictors of recurrence and complications for the endovascular treatment of unruptured middle cerebral artery aneurysm: A high-volume center experience over 12 years. Eur J Radiol. (2023) 163:110833. doi: 10.1016/j.ejrad.2023.110833, PMID: 37080061

[32] Hanel RA Yoon N Sauvageau E Aghaebrahim A Lin E Jadhav AP. Neuroform Atlas Stent for Treatment of Middle Cerebral Artery Aneurysms: 1-Year Outcomes From Neuroform Atlas Stent Pivotal Trial. Neurosurgery. (2021) 89:102–8. doi: 10.1093/neuros/nyab090, PMID: 33826707

[33] Zaidat OO Hanel RA Sauvageau EA Aghaebrahim A Lin E Jadhav AP. Pivotal Trial of the Neuroform Atlas Stent for Treatment of Anterior Circulation Aneurysms: One-Year Outcomes. Stroke. (2020) 51:2087–94. doi: 10.1161/STROKEAHA.119.028418, PMID: 32568654 PMC7306258

[34] Darsaut TE Keough MB Boisseau W Findlay JM Bojanowski MW Chaalala C. Middle Cerebral Artery Aneurysm Trial (MCAAT): A Randomized Care Trial Comparing Surgical and Endovascular Management of MCA Aneurysm Patients. World Neurosurg. (2022) 160:e49–54. doi: 10.1016/j.wneu.2021.12.083, PMID: 34971833

[35] Diestro JDB Li Y Kishore K Omar AT Montanera W Sarma D. A shift from open to endovascular repair in the treatment of ruptured middle cerebral artery aneurysms: a single institution experience. Neuroradiology. (2023) 65:1353–61. doi: 10.1007/s00234-023-03195-w, PMID: 37480480

[36] Xenofontos A Raffalli-Ebezant H Madhavan A Khan H Mastan A Russell I. Simple endovascular coiling: An effective long-term solution for wide-necked ruptured middle cerebral artery aneurysms? A 10-years retrospective study. Neuroradiol J. (2022) 35:573–9. doi: 10.1177/19714009211067406, PMID: 35037769 PMC9513924

[37] Gory B Rouchaud A Saleme S Dalmay F Riva R Caire F. Endovascular treatment of middle cerebral artery aneurysms for 120 nonselected patients: a prospective cohort study. AJNR Am J Neuroradiol. (2014) 35:715–20. doi: 10.3174/ajnr.A3781, PMID: 24200898 PMC7965824

[38] Aydin K Balci S Sencer S Barburoglu M Umutlu MR Arat A. Y-Stent-Assisted Coiling With Low-Profile Neuroform Atlas Stents for Endovascular Treatment of Wide-Necked Complex Intracranial Bifurcation Aneurysms. Neurosurgery. (2020) 87:744–53. doi: 10.1093/neuros/nyz516, PMID: 31807780

[39] Brinjikji W Lanzino G Cloft HJ Rabinstein A Kallmes DF. Endovascular treatment of middle cerebral artery aneurysms: a systematic review and single-center series. Neurosurgery. (2011) 68:397–402. doi: 10.1227/NEU.0b013e318201d7f4, PMID: 21135730

[40] Bracard S Abdel-Kerim A Thuillier L Klein O Anxionnat R Finitsis S. Endovascular coil occlusion of 152 middle cerebral artery aneurysms: initial and midterm angiographic and clinical results. J Neurosurg. (2010) 112:703–8. doi: 10.3171/2009.6.JNS09483, PMID: 19852536

[41] Suzuki S Tateshima S Jahan R Duckwiler GR Murayama Y Gonzalez NR. Endovascular treatment of middle cerebral artery aneurysms with detachable coils: angiographic and clinical outcomes in 115 consecutive patients. Neurosurgery. (2009) 64:876–89. doi: 10.1227/01.NEU.0000343534.05655.37 19287326

[42] Zhang X Zhou Y Zuo Q Duan G Tang H Yang P. Endovascular Treatment of Ruptured Middle Cerebral Artery Aneurysms: A Single-Arm Meta-Analysis and Systematic Review. World Neurosurg. (2019) 127:559–66. doi: 10.1016/j.wneu.2019.01.066, PMID: 30685370

[43] Calvacante T Derrey S Curey S Langlois O Fréger P Gérardin E. Distal middle cerebral artery aneurysm: A proposition of microsurgical management. Neurochirurgie. (2013) 59:121–7. doi: 10.1016/j.neuchi.2013.04.007, PMID: 23806761

[44] Baltacioğlu F Cekirge S Saatci I Oztürk H Arat A Pamir N. Distal middle cerebral artery aneurysms. Endovascular treatment results with literature review. Interv Neuroradiol. (2002) 8:399–407. doi: 10.1177/159101990200800409, PMID: 20594501 PMC3572496

[45] Cagnazzo F di Carlo DT Cappucci M Lefevre PH Costalat V Perrini P. Acutely Ruptured Intracranial Aneurysms Treated with Flow-Diverter Stents: A Systematic Review and Meta-Analysis. AJNR Am J Neuroradiol. (2018) 39:1669–75. doi: 10.3174/ajnr.A5730, PMID: 30049721 PMC7655299

[46] Diestro JDB Adeeb N Dibas M Boisseau W Harker P Brinjikji W. Flow Diversion for Middle Cerebral Artery Aneurysms: An International Cohort Study. Neurosurgery. (2021) 89:1112–21. doi: 10.1093/neuros/nyab365, PMID: 34624100

[47] Soydemir E Gündoğmuş CA Türeli D Andaç Baltacıoğlu N Bayri Y Baltacıoğlu F. Safety and efficacy of flow diverter stents in the treatment of middle cerebral artery aneurysms: a single-center experience and follow-up data. Diagn Intervent Radiol (Ankara, Turkey). (2023) 29:350–8. doi: 10.4274/dir.2022.211050, PMID: 36988000 PMC10679704

[48] Liang F Yang Y Luo L Liao B Zhang G Ou S. Endovascular treatment of complex middle cerebral artery aneurysms using TuBridge flow diverters. Interv Neuroradiol. (2020) 26:539–46. doi: 10.1177/1591019920946216, PMID: 32722987 PMC7645195

[49] Salem MM Khorasanizadeh M Lay SV Renieri L Kuhn AL Sweid A. Endoluminal flow diverting stents for middle cerebral artery bifurcation aneurysms: multicenter cohort. J Neurointerv Surg. (2022) 14:1084–9. doi: 10.1136/neurintsurg-2021-018224, PMID: 34732531

[50] Cagnazzo F Mantilla D Lefevre PH Dargazanli C Gascou G Costalat V. Treatment of Middle Cerebral Artery Aneurysms with Flow-Diverter Stents: A Systematic Review and Meta-Analysis. AJNR Am J Neuroradiol. (2017) 38:2289–94. doi: 10.3174/ajnr.A5388, PMID: 28982785 PMC7963743

[51] Piano M Lozupone E Milonia L Pero G Cervo A Macera A. Flow diverter devices in the treatment of complex middle cerebral artery aneurysms when surgical and endovascular treatments are challenging. J Stroke Cerebrovasc Dis. (2022) 31:106760. doi: 10.1016/j.jstrokecerebrovasdis.2022.106760, PMID: 36201991

[52] Yavuz K Geyik S Saatci I Cekirge HS. Endovascular treatment of middle cerebral artery aneurysms with flow modification with the use of the pipeline embolization device. AJNR Am J Neuroradiol. (2014) 35:529–35. doi: 10.3174/ajnr.A3692, PMID: 24072620 PMC7964741

[53] Cekirge HS Saatci I. A New Aneurysm Occlusion Classification after the Impact of Flow Modification. AJNR Am J Neuroradiol. (2016) 37:19–24. doi: 10.3174/ajnr.A4489, PMID: 26316566 PMC7960201

[54] Lena J Fargen KM. Flow Diversion and Middle Cerebral Artery Aneurysms: Is Successful Aneurysm Occlusion Dependent on Branch Occlusion? World Neurosurg. (2016) 90:630–1. doi: 10.1016/j.wneu.2016.01.006, PMID: 26806067

[55] Iosif C Mounayer C Yavuz K Saleme S Geyik S Cekirge HS. Middle Cerebral Artery Bifurcation Aneurysms Treated by Extrasaccular Flow Diverters: Midterm Angiographic Evolution and Clinical Outcome. AJNR Am J Neuroradiol. (2017) 38:310–6. doi: 10.3174/ajnr.A5022, PMID: 27979794 PMC7963807

[56] Lauzier DC Root BK Kayan Y Almandoz JED Osbun JW Chatterjee AR. Pipeline embolization of proximal middle cerebral artery aneurysms: A multicenter cohort study. Interv Neuroradiol. (2022) 28:50–7. doi: 10.1177/15910199211015578, PMID: 33951971 PMC8905083

[57] Daou B Jabbour P. Flow Diversion for Treating Middle Cerebral Artery Aneurysms. World Neurosurg. (2016) 90:627–9. doi: 10.1016/j.wneu.2016.01.003 26780283

[58] Topcuoglu OM Akgul E Daglioglu E Topcuoglu ED Peker A Akmangit I. Flow Diversion in Middle Cerebral Artery Aneurysms: Is It Really an All-Purpose Treatment? World Neurosurg. (2016) 87:317–27. doi: 10.1016/j.wneu.2015.11.073, PMID: 26723288

[59] Kashkoush A El-Abtah ME Petitt JC Glauser G Winkelman R Achey RL. Flow diversion for the treatment of intracranial bifurcation aneurysms: a systematic review and meta-analysis. J Neurointerv Surg. (2023):20582. doi: 10.1136/jnis-2023-020582, PMID: 37541838

[60] Naamani KE Chen CJ Abbas R Sweid A Sioutas GS Badih K. Woven EndoBridge versus stent-assisted coil embolization of cerebral bifurcation aneurysms. J Neurosurg. (2022) 137:1786–93. doi: 10.3171/2022.3.JNS2217, PMID: 35535832

[61] Fatania K Patankar DT. Comprehensive review of the recent advances in devices for endovascular treatment of complex brain aneurysms. Br J Radiol. (2022) 95:20210538. doi: 10.1259/bjr.20210538, PMID: 34609898 PMC8722252

[62] Hecker C Broussalis E Griessenauer CJ Killer-Oberpfalzer M. A mini-review of intrasaccular flow diverters. J Neurointerv Surg. (2023) 15:70–4. doi: 10.1136/neurintsurg-2021-018426, PMID: 35580985

[63] Dmytriw AA Salem MM Yang VXD Krings T Pereira VM Moore JM. Endosaccular Flow Disruption: A New Frontier in Endovascular Aneurysm Management. Neurosurgery. (2020) 86:170–81. doi: 10.1093/neuros/nyz017, PMID: 30834934 PMC7239377

[64] Lee KB Suh CH Song Y Kwon B Kim MH Yoon JT. Trends of Expanding Indications of Woven EndoBridge Devices for the Treatment of Intracranial Aneurysms: A Systematic Review and Meta-analysis. Clin Neuroradiol. (2023) 33:227–35. doi: 10.1007/s00062-022-01207-5, PMID: 36036257

[65] Pagano P Cortese J Soize S Caroff J Manceau PF Moret J. Aneurysm Treatment with Woven EndoBridge-17: Angiographic and Clinical Results at 12 Months from a Retrospective, 2-Center Series. AJNR Am J Neuroradiol. (2023) 44:467–73. doi: 10.3174/ajnr.A7830, PMID: 36997284 PMC10084902

[66] Fiorella D Molyneux A Coon A Szikora I Saatci I Baltacioglu F. Demographic, procedural and 30-day safety results from the WEB Intra-saccular Therapy Study (WEB-IT). J Neurointerv Surg. (2017) 9:1191–6. doi: 10.1136/neurintsurg-2016-012841, PMID: 28096478

[67] Pierot L Costalat V Moret J Szikora I Klisch J Herbreteau D. Safety and efficacy of aneurysm treatment with WEB: results of the WEBCAST study. J Neurosurg. (2016) 124:1250–6. doi: 10.3171/2015.2.JNS142634, PMID: 26381253

[68] Pierot L Gubucz I Buhk JH Holtmannspötter M Herbreteau D Stockx L. Safety and Efficacy of Aneurysm Treatment with the WEB: Results of the WEBCAST 2 Study. AJNR Am J Neuroradiol. (2017) 38:1151–5. doi: 10.3174/ajnr.A5178, PMID: 28450432 PMC7960101

[69] Pierot L Moret J Turjman F Herbreteau D Raoult H Barreau X. WEB Treatment of Intracranial Aneurysms: Clinical and Anatomic Results in the French Observatory. AJNR Am J Neuroradiol. (2016) 37:655–9. doi: 10.3174/ajnr.A4578, PMID: 26514608 PMC7960156

[70] Pierot L Moret J Turjman F Herbreteau D Raoult H Barreau X. WEB Treatment of Intracranial Aneurysms: Feasibility, Complications, and 1-Month Safety Results with the WEB DL and WEB SL/SLS in the French Observatory. AJNR Am J Neuroradiol. (2015) 36:922–7. doi: 10.3174/ajnr.A4230, PMID: 25655876 PMC7990613

[71] Sabuzi F Cortese J Da Ros V Mihalea C Chalumeau V Moret J. How a decade of aneurysms embolization with the Woven EndoBridge has changed our understanding and practices? J Neuroradiol. (2023) 50:518–22. doi: 10.1016/j.neurad.2023.02.006, PMID: 36868371

[72] Adeeb N Dibas M Diestro JDB Cuellar-Saenz HH Sweid A Kandregula S. Multicenter Study for the Treatment of Sidewall versus Bifurcation Intracranial Aneurysms with Use of Woven EndoBridge (WEB). Radiology. (2022) 304:372–82. doi: 10.1148/radiol.212006, PMID: 35438564

[73] Adeeb N Dibas M Diestro JDB Phan K Cuellar-Saenz HH Sweid A. Comparing treatment outcomes of various intracranial bifurcation aneurysms locations using the Woven EndoBridge (WEB) device. J Neurointerv Surg. (2023) 15:558–65. doi: 10.1136/neurintsurg-2022-018694, PMID: 35483912

[74] Al Saiegh F Velagapudi L Khanna O Sweid A Mouchtouris N Baldassari MP. Predictors of aneurysm occlusion following treatment with the WEB device: systematic review and case series. Neurosurg Rev. (2022) 45:925–36. doi: 10.1007/s10143-021-01638-7, PMID: 34480649

[75] Goertz L Liebig T Siebert E Dorn F Pflaeging M Forbrig R. Long-term clinical and angiographic outcome of the Woven EndoBridge (WEB) for endovascular treatment of intracranial aneurysms. Sci Rep. (2022) 12:11467. doi: 10.1038/s41598-022-14945-w, PMID: 35794159 PMC9259699

[76] Pierot L Szikora I Barreau X Holtmannspoetter M Spelle L Klisch J. Aneurysm treatment with the Woven EndoBridge (WEB) device in the combined population of two prospective, multicenter series: 5-year follow-up. J Neurointerv Surg. (2023) 15:552–7. doi: 10.1136/neurintsurg-2021-018414, PMID: 35803731 PMC10314010

[77] Cortese J Caroff J Chalumeau V Gallas S Ikka L Moret J. Determinants of cerebral aneurysm occlusion after embolization with the WEB device: a single-institution series of 215 cases with angiographic follow-up. J Neurointerv Surg. (2023) 15:446–51. doi: 10.1136/neurintsurg-2022-018780, PMID: 35428742

[78] Dmytriw AA Diestro JDB Dibas M Phan K Sweid A Cuellar-Saenz HH. International Study of Intracranial Aneurysm Treatment Using Woven EndoBridge: Results of the WorldWideWEB Consortium. Stroke. (2022) 53:e47–9. doi: 10.1161/STROKEAHA.121.037609, PMID: 34915737 PMC8792251

[79] Caroff J Janot K Soize S Marnat G Cortese J Mihalea C. Management of aneurysmal recurrence after Woven EndoBridge (WEB) treatment. J Neurointerv Surg. (2022) 15:939–42. doi: 10.1136/jnis-2022-019645 36288976

[80] Srinivasan VM Dmytriw AA Regenhardt RW Vicenty-Padilla J Alotaibi NM Levy E. Retreatment of Residual and Recurrent Aneurysms After Embolization With the Woven EndoBridge Device: Multicenter Case Series. Neurosurgery. (2022) 90:569–80. doi: 10.1227/neu.0000000000001883, PMID: 35244028 PMC9524592

[81] Akhunbay-Fudge CY Deniz K Tyagi AK Patankar T. Endovascular treatment of wide-necked intracranial aneurysms using the novel Contour Neurovascular System: a single-center safety and feasibility study. J Neurointerv Surg. (2020) 12:987–92. doi: 10.1136/neurintsurg-2019-015628, PMID: 31974281 PMC7509519

[82] Ghozy S Lashin BI Elfil M Bilgin C Kobeissi H Shehata M. The safety and effectiveness of the Contour Neurovascular System for the treatment of wide-necked aneurysms: A systematic review and meta-analysis of early experience. Interv Neuroradiol. (2022):159101992211395. doi: 10.1177/15910199221139546 PMC11483783 36384322

[83] Liebig T Killer-Oberpfalzer M Gal G Schramm P Berlis A Dorn F. The Safety and Effectiveness of the Contour Neurovascular System (Contour) for the Treatment of Bifurcation Aneurysms: The CERUS Study. Neurosurgery. (2022) 90:270–7. doi: 10.1227/NEU.0000000000001783, PMID: 35113830

[84] Biondi A Primikiris P Vitale G Charbonnier G. Endosaccular flow disruption with the Contour Neurovascular System: angiographic and clinical results in a single-center study of 60 unruptured intracranial aneurysms. J Neurointerv Surg. (2022) 15:838–43. doi: 10.1136/jnis-2022-019271 35995545

[85] Diana F de Dios LM Peschillo S Raz E Yoshimura S Requena Ruiz M. Intrasaccular Flow Disruptor-Assisted Coiling of Intracranial Aneurysms Using the Novel Contour Neurovascular Systems and NEQSTENT: A Single-Center Safety and Feasibility Study. Brain Sci. (2022) 12:991. doi: 10.3390/brainsci12080991, PMID: 35892432 PMC9394360

[86] Piotin M Biondi A Sourour N Mounayer C Jaworski M Mangiafico S. The LUNA aneurysm embolization system for intracranial aneurysm treatment: short-term, mid-term and long-term clinical and angiographic results. J Neurointerv Surg. (2018) 10:e34. doi: 10.1136/neurintsurg-2018-013767, PMID: 29669856 PMC6288707

[87] Piotin M Fahed R Redjem H Smajda S Desilles JP Escalard S. The ARTISSE intrasaccular device for intracranial aneurysm treatment: short-term, mid-term and long-term clinical and angiographic results. J Neurointerv Surg. (2022) 14:957–61. doi: 10.1136/neurintsurg-2021-017806, PMID: 34611032

[88] Sirakov A Aguilar-Perez M AlMatter M Henkes H. Complex Wide-necked and lobulated aneurysm of the middle cerebral artery bifurcation: treatment with a pconus2 neck bridging device and p48mw flow modulation device. Clin Neuroradiol. (2020) 30:633–7. doi: 10.1007/s00062-019-00862-5, PMID: 31807809 PMC7471178

[89] Guenego A Mine B Bonnet T Elens S Vazquez Suarez J Jodaitis L. Long-term follow-up of the pCONus device for the treatment of wide-neck bifurcation aneurysms. Interv Neuroradiol. (2022) 28:455–62. doi: 10.1177/15910199211040279, PMID: 34516326 PMC9326855

[90] Gory B Aguilar-Pérez M Pomero E Turjman F Weber W Fischer S. One-year Angiographic Results After pCONus Stent-Assisted Coiling of 40 Wide-Neck Middle Cerebral Artery Aneurysms. Neurosurgery. (2017) 80:925–33. doi: 10.1093/neuros/nyw131, PMID: 28368544

[91] Morales-Caba L Lylyk I Vázquez-Añón V Bleise C Scrivano E Perez N. The pCONUS2 and pCONUS2 HPC neck bridging devices: results from an international multicenter retrospective study. Clin Neuroradiol. (2023) 33:129–36. doi: 10.1007/s00062-022-01191-w, PMID: 35819477 PMC10014770

[92] Yeomans J Gatt S Habeeb Mohamed E Crossley R Keston P Minks D. pCONUS 2 and pCONUS 2-HPC in the treatment of wide-necked intracranial aneurysms: Multicentre UK experience. Interv Neuroradiol. (2023):159101992211504. doi: 10.1177/15910199221150467 PMC11833899 36617807

[93] Ulfert C Pfaff J Schönenberger S Bösel J Herweh C Pham M. The pCONus device in treatment of wide-necked aneurysms: technical and midterm clinical and angiographic results. Clin Neuroradiol. (2018) 28:47–54. doi: 10.1007/s00062-016-0542-z, PMID: 27637921

[94] Aguilar Pérez M Henkes H Kurre W Bleise C Lylyk PN Lundquist J. Results of the pToWin Study: Using the pCONUS Device for the Treatment of Wide-Neck Intracranial Aneurysms. J Clin Med. (2022) 11:884. doi: 10.3390/jcm11030884, PMID: 35160333 PMC8836830

[95] Mühl-Benninghaus R Simgen A Reith W Yilmaz U. The Barrel stent: new treatment option for stent-assisted coiling of wide-necked bifurcation aneurysms-results of a single-center study. J Neurointerv Surg. (2017) 9:1219–22. doi: 10.1136/neurintsurg-2016-012718, PMID: 27856649

[96] Gory B Blanc R Turjman F Berge J Piotin M. The Barrel vascular reconstruction device for endovascular coiling of wide-necked intracranial aneurysms: a multicenter, prospective, post-marketing study. J Neurointerv Surg. (2018) 10:969–74. doi: 10.1136/neurintsurg-2017-013602, PMID: 29437935 PMC6166605

[97] Kabbasch C Mpotsaris A Maus V Altenbernd JC Loehr C. The barrel vascular reconstruction device: a retrospective, observational multicentric study. Clin Neuroradiol. (2019) 29:295–301. doi: 10.1007/s00062-017-0660-2, PMID: 29318353

[98] De Vries J Boogaarts HD Sørensen L Holtmannspoetter M Benndorf G Turowski B. eCLIPs bifurcation remodeling system for treatment of wide neck bifurcation aneurysms with extremely low dome-to-neck and aspect ratios: a multicenter experience. J Neurointerv Surg. (2021) 13:438–42. doi: 10.1136/neurintsurg-2020-016354, PMID: 32788388 PMC8053345

[99] de Vries J Aquarius R Sørensen L Boogaarts HD Turowski B van Zwam WH. Safety and efficacy of the eCLIPs bifurcation remodelling system for the treatment of wide necked bifurcation aneurysms: 1 year results from the European eCLIPs Safety, Feasibility, and Efficacy Study (EESIS). J Neurointerv Surg. (2023) 15:163–71. doi: 10.1136/neurintsurg-2021-018460, PMID: 35393338

[100] Diestro JDB Keough MB Ashforth RA Chow MM Rempel JL Marotta TR. Treatment of wide-necked bifurcation aneurysms with the eCLIPs device: 5-year experience of a single center. J Neurointerv Surg. (2023) 15:461–4. doi: 10.1136/neurintsurg-2022-018731, PMID: 35545426

